# Role of emmprin in endometrial cancer

**DOI:** 10.1186/1471-2407-12-191

**Published:** 2012-05-28

**Authors:** Keiichiro Nakamura, Junichi Kodama, Atsushi Hongo, Yuji Hiramatsu

**Affiliations:** 1 Department of Obstetrics and Gynecology, Dentistry and Pharmaceutical Sciences, Okayama University Graduate School of Medicine, 2-5-1 Shikata-cho, Kita-ku, Okayama, 700-8558, Japan

**Keywords:** Endometrial cancer, Emmprin, Epithelial-mesenchymal transition, Predictor of favorable prognosis, Potential therapeutic target

## Abstract

**Background:**

Extracellular matrix metalloproteinase inducer (Emmprin/CD147) is a transmembrane glycoprotein that belongs to the immunoglobulin superfamily. Enriched on the surface of many tumor cells, emmprin promotes tumor growth, invasion, metastasis and angiogenesis. We evaluated the clinical importance of emmprin and investigated its role in endometrial cancer.

**Methods:**

Emmprin expression was examined in uterine normal endometrium, endometrial hyperplasia and cancer specimens by immunohistochemistry. In addition, the biological functions and inhibitory effects of an emmprin knockdown were investigated in HEC-50B and KLE endometrial cancer cell lines.

**Results:**

The levels of emmprin expression were significantly increased in the endometrial cancer specimens compared with the normal endometrium and endometrial hyperplasia specimens (*p* < 0.05). The disease-free survival (DFS) and overall survival (OS) rates of patients with high emmprin expression were significantly higher than those of patients with low emmprin expression (DFS: *p* < 0.001; OS: *p* < 0.001). Emmprin knockdown by the siRNA led to cell proliferation, migration and invasion through TGF-β, EGF, NF-κB, VEGF, MMP-2, and MMP-9 expression, which in turn resulted in increased levels of E-cadherin and reduced levels of Vimentin and Snail in endometrial cancer.

**Conclusions:**

The present findings suggest that low emmprin expression might be a predictor of favorable prognosis in endometrial cancer patients, and that emmprin may represent a potential therapeutic target for endometrial cancer.

## Background

Endometrial carcinoma is the most common gynecologic malignancy in the United States, with an estimated 43,470 new cases diagnosed in 2010 [1]. In Japan, endometrial cancer is currently the fourth most common gynecologic malignancy in women, with an estimated incidence of 6,665 new cases in 2010 [2]. Clinical parameters such as the disease stage, nuclear grade, histologic subtypes and tumor size are correlated with the outcome of the disease. Therefore, it is hoped that a better understanding of the molecular mechanism underlying the progression of endometrial cancer will lead to new insights for novel therapeutic targets.

Extracellular matrix metalloproteinase inducer (Emmprin) is a highly glycosylated transmembrane protein that belongs to the immunoglobulin superfamily and is encoded by a gene localized to 19p13.3. [3,4]. Most cell types show constitutive expression of low levels of emmprin owing to its involvement in many physiologic processes, while high levels of emmprin expression are observed during remodeling processes such as inflammation, embryonic development, wound healing and tumor progression [5-9]. Emmprin has recently been recognized as an important modulator of tumor-stromal communication and mediates a wide range of tumor-promoting molecular events, including the acquisition of anchorage-independent growth and invasive phenotype by tumor cells and tumor cell-induced angiogenesis [10]. To date, several studies on emmprin expression in tumor tissues have been published. Emmprin is overexpressed in a variety of human cancers, and its overexpression is correlated with clinically aggressive behavior and poor patient survival [11-19].

The function of emmprin in malignant tumors has been investigated in many different experimental systems. Emmprin on tumor cells stimulates peritumoral fibroblasts or cancer cells to secrete increased amounts of matrix metalloproteinases (MMPs), which are responsible for degradation of the extracellular matrix (ECM), and therefore facilitate tumor invasion and metastasis [4,15]. Emmprin can promote tumor cell invasion via activation of urokinase-type plasminogen activator, nuclear factor kappa B (NF-κB) and c-Jun N-terminal kinase (JNK) and stimulate tumor angiogenesis via vascular endothelial cell growth factor (VEGF), and is thus implicated in other aspects of tumor progression, such as tumor survival and angiogenesis [20,21].

Epithelial-mesenchymal transition (EMT) is an essential morphologic conversion that occurs during embryonic development. Increasing evidence suggests that a similar process occurs during cancer progression when tumor cells acquire the capacity to migrate, invade and metastasize [22]. Loss of cell-cell adhesion is a prerequisite for EMT and involves functional loss of E-cadherin. The activation of E-cadherin repressors may be induced by signaling mediated by growth factors, including transforming growth factor (TGF)-β, insulin-like growth factor (IGF), epidermal growth factor (EGF) and tumor necrosis factor (TNF)-α, via their specific receptors [23-25]. The transcriptional repressors of E-cadherin are zinc finger transcription factors, including Snail, Slug, Smad-interacting protein 1 (SIP1) and Twist, which is also implicated in this process [26-29]. However, it remains unclear how the mechanisms of action of emmprin are involved in endometrial cancer and how its roles are intertwined.

In the present study, we examined whether emmprin expression is correlated with clinicopathological characteristics in patients suffering from endometrial cancer. The main aim of this study was to determine whether emmprin could represent a potential therapeutic target for endometrial cancer.

## Methods

### Patients and tissue specimens

Patients with normal endometrium (n = 20), endometrial hyperplasia (n = 10) (simple, n = 1; complex, n = 2; atypical complex, n = 7), adenocarcinoma and carcinosarcoma (n = 134) were treated at Okayama University Hospital between January 2000 and November 2009. All of the patients underwent a total abdominal hysterectomy, bilateral salpingo-oophorectomy and partial omentectomy with or without pelvic and/or para-aortic lymphadenectomy. Pelvic lymph node dissection included the right and left common iliac, external iliac, suprainguinal, internal iliac, obturator, sacral and parametrial nodal chains. Para-aortic lymph node dissection included the nodes located from the bifurcation of the aorta to the level of the renal vein (n = 36). Adjuvant chemotherapy was used depending the FIGO stage, grade, patient preference and physician discretion. Our standard chemotherapy consisted of paclitaxel (175 mg/m^2^ infused over 3 h) and carboplatin (dosed for an area under the curve of 5) for 3–6 cycles (n = 52). Patients with neoadjuvant chemotherapy were excluded from this study. Tumor specimens were obtained at the time of surgery, immediately fixed in 10% neutral-buffered formalin and embedded in paraffin. The histological cell types were diagnosed according to the WHO classification, and all 134 cancer specimens were classified as endometrioid adenocarcinomas and carcinosarcoma. The histological grades were assigned according to the International Federation of Gynecology and Obstetrics (FIGO) staging classification as follows: grade 1, n = 51; grade 2, n = 55; grade 3, n = 22; and carcinosarcoma, n = 6) The surgical stages were reviewed based on the FIGO staging system as follows: stage I, n = 74; stage II, n = 11; stage III, n = 39; stage IV, n = 10. The median age at the time of surgery was 57.7 years (range, 28–85). The disease-free survival (DFS) and overall survival (OS) rates were defined as the interval between the initial operation and clinically or radiologically proven recurrence or death, respectively. This study was approved by the Institutional Review Board of Okayama University Hospital.

### Immunohistochemistry and staining evaluation

Formalin-fixed paraffin-embedded sections (4-μm thickness) were deparaffinized with xylene and rehydrated in ethanol. Endogenous peroxidase activity was quenched by treatment with methanol containing 0.3% hydrogen peroxidase for 15 min. The sections were then incubated with an anti-emmprin antibody (Santa Cruz Biotechnology, Santa Cruz, CA) at room temperature, followed by staining with a streptavidin-biotin -peroxidase kit (Nichirei, Tokyo, Japan). The sections were counterstained with hematoxylin. The levels of emmprin staining in epithelial cells were classified into three groups by scoring the percentages of positive cells: 2, strong, >50% of cells stained; 1, moderate, 10–50% of cells stained; 0, weak, <10% of cells stained. Microscopic analyses were independently conducted by two independent examiners with no prior knowledge of the clinical data. Final decisions in controversial cases were made using a conference microscope.

### Cell culture, media and small-interfering RNA (siRNA) transfection

The HEC-50B (Japanese Collection of Research Bioresources (JCRB) number: JCRB1145) and KLE (ATCC number: CRL-1622) cell lines evaluated were derived from human endometrial cancers. The HEC-50B cell line was maintained in Dulbecco’s modified eagle’s medium (DMEM) (Life Technologies, Grand Island, NY) supplemented with 10% fetal bovine serum (FBS). The KLE cell line was maintained in DMEM/F12 medium (Life Technologies, Grand Island, NY) supplemented with 10% FBS. HEC-50B and KLE cells were trypsinized and plated in culture dishes. At ~50% confluency, the cells were transfected with an annealed emmprin siRNA (sc-35298) or scramble siRNA as an empty vector for gene silencing (final concentration, 100 nmol/L) using a siRNA transfection reagent (sc-29528; Santa Cruz Biotechnology). The cells were cultured with each siRNA for 24, 48 and 72 h.

### Western blotting analysis

Cell lysates were collected and estimated using a Protein Assay system (Bio-Rad, Hercules, CA) according to the manufacturer’s protocols. Proteins from each cell line were subjected to SDS-PAGE, and then transferred onto nitrocellulose membranes. The polyclonal and monoclonal antibodies used for immunoblotting were as follows: anti-emmprin, anti-NF-κB p65 and anti-NF-κB phospho-p65 (p-p65) (Santa Cruz Biotechnology, Santa Cruz, CA); and anti-β-actin (Sigma Chemical Co., St. Louis, MO). The working dilution of all the primary antibodies was 1:1000. The membranes were then incubated with appropriate secondary antibodies. The resulting antigen-antibody complexes were detected with an enhanced chemiluminescence kit (Amersham Biosciences, Piscataway, NJ).

### MTS assay and cell viability assay

To evaluate the effects of emmprin on cell proliferation, MTS and cell viability assays were performed. For the MTS assay, cells were seeded into 96-well plates and transfected when the cell density reached 5 × 10^4^ cells/well. After transfection of the cells with the emmprin siRNA for 24, 48 and 72 h, MTS (Promega, Madison, WI) was added for 2 h. The absorbances were measured at 490 nm using an ELISA plate-reader (Bio-Rad Systems, Hercules, CA). The cell viability was analyzed using SYTO 10 green fluorescent nucleic acid stain and dead red (ethidium homodimer-2) nucleic acid stain (Live/Dead^®^ reduced biohazard viability/cytotoxicity kit; Invitrogen, Eugene, OR). Briefly, cells were transfected with the emmprin siRNA for 72 h and then incubated with SYTO 10 green fluorescent nucleic acid stain and dead red nucleic acid stain for 15 min. The cell fluorescence was observed using a fluorescence microscope (Olympus, Tokyo, Japan).

### Motility and invasion assay

To investigate the effects of the emmprin siRNA on cell motility and invasion, we used monolayer wounding (scratch) and chemotaxicells (polycarbonate filter, pore size 8 μm; Kurabo, Osaka, Japan) coated with type IV collagen. For the monolayer wounding assay, cells were allowed to form a monolayer on a culture dish, and a wound was made by scratching the monolayer with a pipette tip. After removal of the scratched cells and transfection with the emmprin siRNA, the cells were cultured for 24 h. For the chemotaxicells coated with type IV collagen, cells (1 × 10^5^) in 100 μL of DMEM/F12 medium or DMEM supplemented with 0.1% bovine serum albumin were placed in the upper compartment, transfected with the emmprin siRNA and then incubated for 72 h. After the incubation, the cells on the upper surface of the filter were wiped off with a cotton swab. The cells on the lower surface were stained with hematoxylin and counted in 10 randomly selected fields.

### Matrigel invasion assay

To investigate the differences in the Matrigel invasion abilities among cells expressing emmprin, we used BD BioCoat Matrigel Invasion Chambers (BD Bioscience, Bedford, MA). HEC-50B and KLE cells transfected with the emmprin siRNA were added *in situ* with 10 μg/mL of DiI (Invitrogen) in DMEM/F12 or DMEM supplemented with 10% FBS for 1 h. Cells (5 × 10^4^) of each genotype were added to the inserts, and 0.75 mL of medium was added to the bottom of each well. After 72 h of incubation, the membranes were removed from the insert and mounted on slides, and the numbers of invading cells were counted under a microscope. The Matrigel assays were performed in triplicate.

### Real-time and quantitative RT-PCR

Total RNA was extracted from the cell lines using an acid guanidinium –phenol -chloroform method (ISOGEN; Nippon Gene, Tokyo, Japan) according to the manufacturer's instructions. Real-time RT-PCR was performed using a LightCycler rapid thermal cycler instrument (Roche Diagnostics, Mannheim, Germany) under the conditions recommended by the manufacturer. The real-time RT-PCR used primers for emmprin, EGF and TGF-β as previously described [30,31]. The PCR products were checked by melting point analyses and their electrophoretic mobilities. Standard curves for calculation of the numbers of transcripts were created using plasmids containing the respective amplified fragments as inserts, and were adjusted to use glyceraldehyde-3-phosphate dehydrogenase (GAPDH) as a reference gene. In addition, the PCR products were analyzed by 1.5% agarose gel electrophoresis. As an internal control, GAPDH mRNA was also measured by quantitative RT-PCR. The quantitative RT-PCR used primers for MMP-2, MMP-9, VEGF, E-cadherin, Vimentin, Snail and GAPDH as described previously [32].

### Transient transfection assay

pNF-κB-responsive and pAP-1-responsive elements were used for NF-κB and AP-1 signaling reporter assays, respectively. pNF-κB-Luc and pAP-1-Luc were purchased from Clontech (Palo Alto, CA). Transient transfections were performed using Lipofectamine™ 2000 reagent (Life Technologies). For the luciferase reporter assays, cells were transfected with 0.5 μg of NF-κB-responsive plasmid, AP-1-responsive plasmid, estrogen-responsive plasmid or progesterone-responsive plasmid in combination with 0.05 μg of pTK-RLUC (Promega) as an internal control. Their proteins were extracted using a Dual-Luciferase reporter assay system (Promega). The firefly and Renilla luciferase activities were measured concurrently for 12 sec using a luminometer (LUMAT LB9507; Berthold, Wildbad, Germany). The assays were carried out for quadruplicate transfection experiments, and at least three independent values were analyzed to confirm reproducibility.

### Cell growth in monolayers

For evaluation of cell growth in monolayers, cells were plated at a density of 3 × 10^4^ cells/well in 6-well plates containing DMEM or DMEM/F12 supplemented with 10% FBS. The cell numbers were counted in triplicate after 1, 3, 5 and 7 days using a hemocytometer to assess cell proliferation.

### Statistical analysis

Statistical analyses were performed using the Mann–Whitney *U*-test for comparisons with controls and one-factor ANOVA followed by Fisher's protected least significance difference test for all pairwise comparisons. The survival rates were calculated by the Kaplan–Meier method, and the differences between the survival curves were examined by using the log-rank test. The analyses were performed with the software package StatView version 5.0 (Abacus Concepts, Berkeley, CA). Differences were considered significant at *p* < 0.05.

## Results

### The expression levels of emmprin were examined in human endometrial tissues by immunostaining

**Figure 1:**
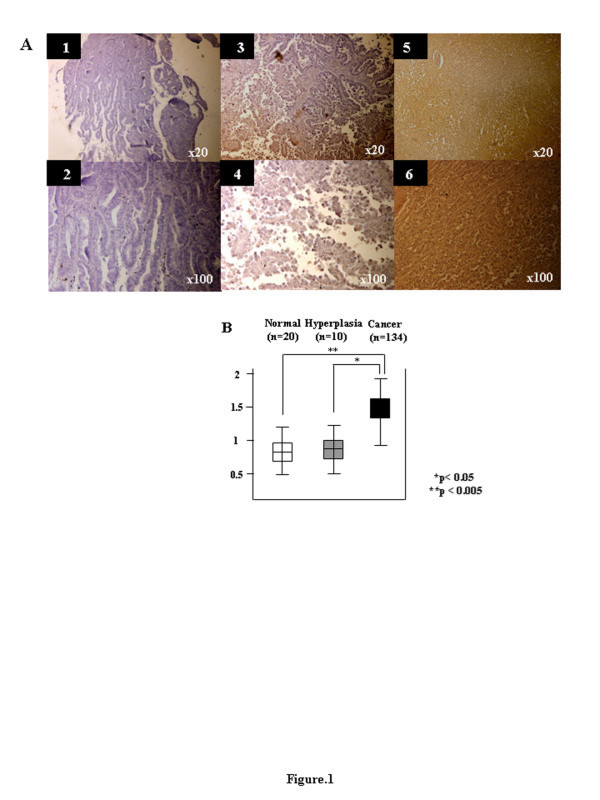
**Representative immunostaining patterns of emmprin. A**) 1. Weak epithelial cell staining (original magnification x20). 2. Weak epithelial cell staining (original magnification x100) (Grade 1 endometrioid adenocarcinoma). 3. Moderate epithelial cell staining (original magnification x20). 4. Moderate epithelial cell staining (original magnification x100) (Grade 2 endometrioid adenocarcinoma). 5. Strong epithelial cell staining (original magnification x20). 6. Strong epithelial cell staining (original magnification x100) (Grade 3 endometrioid adenocarcinoma). **B**) Histograms of emmprin expression according to the specimens (uterine normal endometrium, endometrial hyperplasia and endometrial cancer).

Emmprin-dependent activation may have a role in conventional endometrial tumorigenesis, and therefore the emmprin activation machinery might be important. The expression levels of emmprin were examined in human endometrial tissues by immunostaining. Figure 1 show representative immunostaining patterns of emmprin. Weak epithelial staining was observed in 25 cases (15.2%), moderate staining in 64 cases (38.9%) and strong staining in 76 cases (45.9%). The mean scores of the epithelial staining for emmprin were 0.85 for normal human endometrium, 0.9 for hyperplasia and 1.42 for cancer samples. Interestingly, endometrial cancer had the strongest emmprin expression compared with normal human endometrium and endometrial hyperplasia (*p* < 0.05, Mann–Whitney *U*-test) (Figure 1C). 

**Figure 2:**
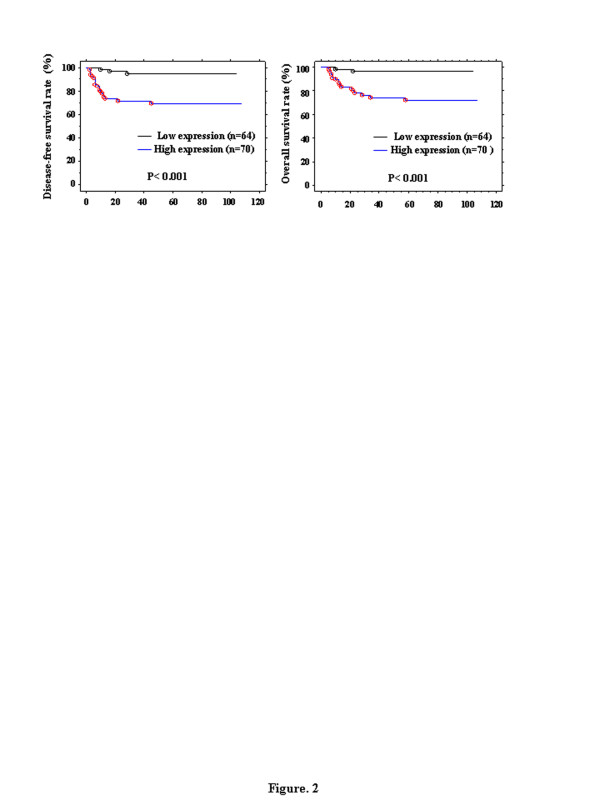
**Disease-free survival (DFS) and overall survival (OS) rates of the 134 patients with endometrial cancer according to the emmprin expression status.** Low epithelial expression, scores 0–1; high epithelial expression, score 2.

Table 1 shows the distribution of cases scored as positive for each of the biological parameters examined, according to the clinicopathological characteristics in the overall population. As expected, the expression of emmprin had significant associations with clinicopathological parameters such as FIGO stage (*p* = 0.009), histology (*p* = 0.017), depth of myometrial invasion (*p* = 0.001), cervical involvement (*p* = 0.001), lymph node metastasis (*p* < 0.001), lymph vascular space (LVS) involvement (*p* < 0.001) and peritoneal cytology (*p* = 0.031), whereas age and ovarian metastasis did not have significant associations (*p* < 0.05, Mann–Whitney *U*-test). Emmprin was most significantly associated with the DFS and OS rates among the prognostic factors using the log-rank test for endometrial cancer. Figure 2 shows the DFS and OS curves of the 134 patients with endometrial cancer, according to the emmprin expression status. The DFS and OS rates of patients with high emmprin expression (score 2) were significantly higher than those of patients with low emmprin expression (scores 0–1) (DFS: *p* < 0.001; OS: *p* < 0.001). Furthermore, we examined independent prognostic factor for DFS and OS of the clinicopathologic factors including stage, histology, lymph node metastasis, deep myometrial invasion, ovarian metastasis and emmprin by multiple analysis. The ovarian metastasis was strongest independent prognostic factor for DFS and OS by multiple analysis (P = 0.0245 and P = 0.0222). However, emmprin expression was not an independent prognostic factor for DFS and OS. 

**Table 1:** Associations of emmprin with clinical factors in endometrial cancer patients

		**Emmprin**	
** Variable**	**Numbers**	**mean ± SE**	***p-*value**
Age (years)			0.445
<60	80	1.38 ± 0.70	
≥60	54	1.48 ± 0.84	
FIGO stage			0.009*
I-II	85	1.30 ± 0.65	
III-IV	49	1.61 ± 0.67	
Histology			0.017*
Type I (G1 + G2)	106	1.34 ± 0.69	
Type II (G3 + carcinosarcoma)	28	1.68 ± 0.54	
Depth of myometrial invasion			0.001*
<1/2	87	1.28 ± 0.66	
≥1/2	47	1.66 ± 0.63	
Cervical involvement			0.001*
Negative	105	1.32 ± 0.67	
Positive	29	1.75 ± 0.57	
Lymph node metastasis			<0.001*
Negative	116	1.34 ± 0.67	
Positive	18	1.89 ± 0.47	
LVS involvement			<0.001*
Negative	90	1.27 ± 0.67	
Positive	44	1.70 ± 0.59	
Ovarian metastasis			0.127
Negative	119	1.38 ± 0.66	
Positive	15	1.66 ± 0.72	
Peritoneal cytology			0.031*
Negative	105	1.35 ± 0.67	
Positive	29	1.65 ± 0.61	

*Mann–Whitney *U*-test; FIGO, International Federation of Gynecology and Obstetrics; LVS, lymph vascular space.

### Protein expression, cell proliferation and cell viability after emmprin siRNA transfection

**Figure 3:**
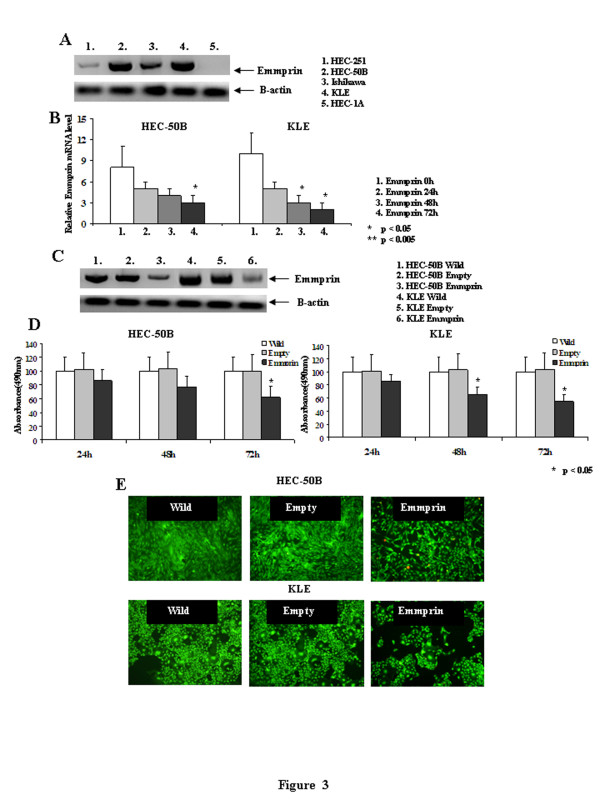
**Emmprin expression in endometrial cancer and real-time PCR, western blot, MTS assays and Cell viabilities of HEC-50B and KLE cells after transfection with the emmprin siRNA. A**) Western blot analysis of emmprin expression in endometrial cancer cell lines (KLE, Ishikawa, HEC-251, HEC-1A and HEC-50B). An anti-β-actin antibody was used after transfection with the emmprin siRNA. **B**) Real-time PCR of emmprin expression after transfection of the emmprin siRNA into HEC-50B and KLE cells for 24, 48 and 72 h. **C**) Western blot analysis of emmprin expression after transfection of the emmprin siRNA into HEC-50B and KLE cells for 72 h. An anti-β-actin antibody was used as a loading control in the same membrane. **D**) MTS assays of the cell proliferation of HEC-50B and KLE cells after transfection with the emmprin siRNA for 24, 48 and 72 h. The assays were carried out for quadruplicate transfection experiments. **E**) Cell viabilities of HEC-50B and KLE cells after transfection with the emmprin siRNA for 72 h evaluated by fluorescence microscopy.

We first examined the protein levels of emmprin in human endometrial cancer cell lines. As shown in Figure 3A, emmprin protein was highly expressed in KLE, HEC-50B and Ishikawa cells, weakly expressed in HEC-251 cells and absent in HEC-1A cells. Among the endometrial cancer cell lines, we chose KLE and HEC-50B endometrial cancer cells for further study. Use of the emmprin siRNA to trigger differentiation in endometrial cancer cells led to a time-dependent reduction in the emmprin mRNA that peaked around 72 h (Figure 3B). The expression of emmprin protein was significantly decreased in KLE and HEC-251 cells after transfection with the emmprin siRNA for 72 h (Figure 3C). The cell proliferation of HEC-50B and KLE cells transfected with the emmprin siRNA for 72 h was significantly inhibited as evaluated by MTS assays (Figure 3D). The cell viabilities of HEC-50B and KLE cells were evaluated after transfection with the emmprin siRNA. The percentages of viable cells were decreased to 72.1% (HEC-50B) and 53.6% (KLE) of the wild-type cell viabilities after 72 h of transfection with the emmprin siRNA (Figure 3E). 

### Motility, invasiveness and matrigel invasion after emmprin siRNA transfection

**Figure 4:**
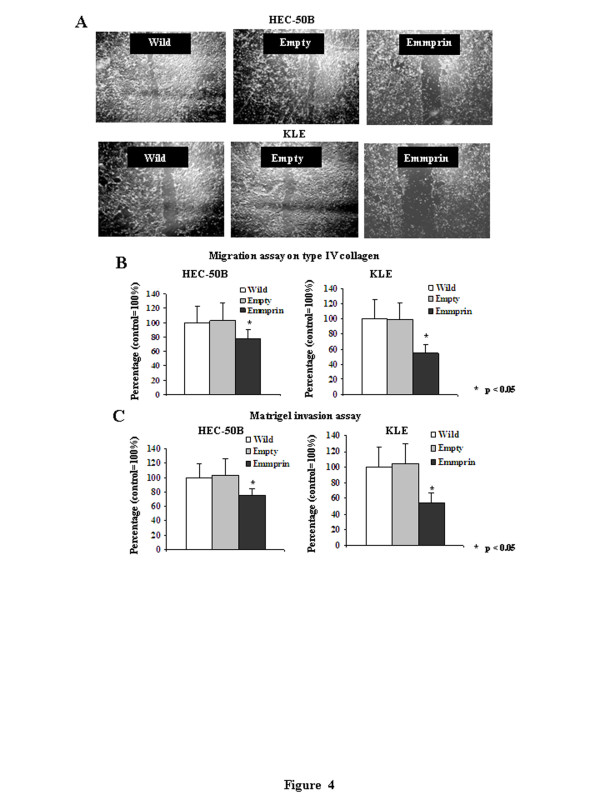
**Effects on the motility and matrigel invasion of HEC-50B and KLE cells after transfection with the emmprin siRNA. A**) Cell scratch assays of HEC-50B and KLE cells after transfection with the emmprin siRNA for 24 h. **B**) Migration assays on type IV collagen for HEC-50B and KLE cells after transfection with the emmprin siRNA for 72 h. **C**) Matrigel invasion assays after transfection of the emmprin siRNA into HEC-50B and KLE cells for 72 h. Following the incubation, the membranes were removed from the inserts and mounted on slides. The numbers of invading cells were counted under a microscope. The motility and Matrigel assays were performed in triplicate.

The purpose of these experiments was to study the role of endometrial cancers in the motility, invasiveness and Matrigel invasion of cells transfected with the emmprin siRNA. The cells transfected with the emmprin siRNA were much slower than cells transfected with the wild-type or empty vector in the motility and invasiveness assay (Figures 4A and 4B). These findings suggest that emmprin is associated with cell adhesion and spreading. The percentages of cells reaching the bottom of the filter were decreased to 61.5% and 48.9% in the Matrigel invasion assay after transfection of the emmprin siRNA for 72 h into HEC-50B and KLE cells, respectively (Figure 4C). 

### EMT after emmprin siRNA transfection

**Figure 5:**
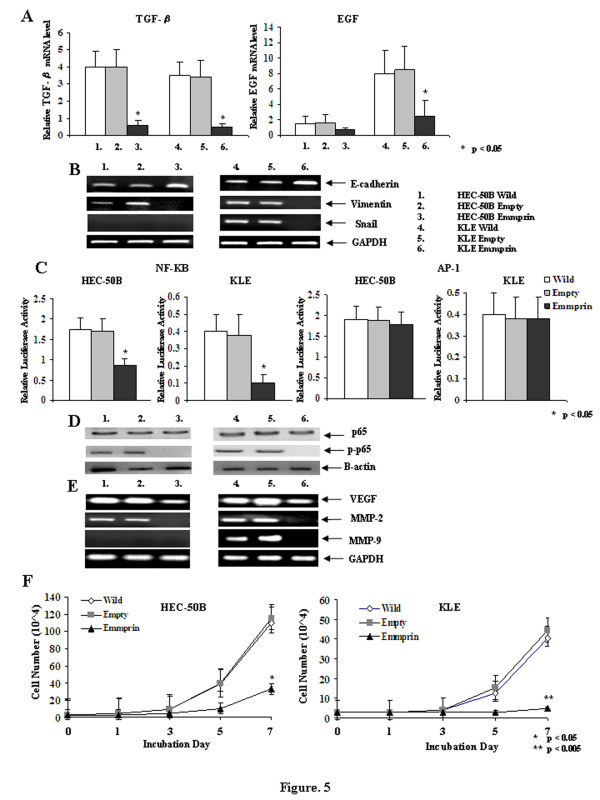
**Real-time RT-PCR, quantitative RT-PCR, transient transfection assays, western blotting and cell growth of HEC-50B and KLE cells after transfection with the emmprin siRNA. A**) Real-time PCR of TGF-β and EGF expression after transfection of the emmprin siRNA into HEC-50B and KLE cells for 72 h. **B**) Quantitative PCR analyses of E-cadherin, Vimentin and Snail expression levels after transfection of the emmprin siRNA into HEC-50B and KLE cells for 72 h. GAPDH was used as a loading control. **C**) Transient transfection assays of NF-κB and AP-1 into HEC-50B and KLE cells after transfection with the emmprin siRNA for 72 h. The assays were carried out for quadruplicate transfection experiments. **D**) Western blot analysis of NF-κB p65 and p-p65 expression after transfection of the emmprin siRNA into HEC-50B and KLE cells for 72 h. An anti-β-actin antibody was used as a loading control in the same membrane. **E**) PCR analyses of VEGF, MMP-2 and MMP-9 expression levels after transfection of the emmprin siRNA into HEC-50B and KLE cells for 72 h. GAPDH was used as a loading control. **F**) Cell growth in monolayers after transfection of the emmprin siRNA. The monolayer growth was evaluated for HEC-50B and KLE cells transfected with the emmprin siRNA and cultured for 1, 3, 5 and 7 days in DMEM or DMEM/F12 medium supplemented with 10% FBS. The numbers represent the data from triplicate experiments.

MMP activities have been implicated in EMT by activating growth factors and their receptors and cleaving cell-cell and cell-ECM adhesion molecules. We examined the transcriptional repressors of EMT families and each growth factor. The expression of TGF-β and EGF were decreased after transfection of the emmprin siRNA into HEC-50B and KLE cells (Figure 5A). Loss of cell-cell adhesion is a prerequisite for EMT and involves functional loss of E-cadherin. Transfection of the emmprin siRNA caused significant increases in the expression of E-cadherin in HEC-50B and KLE cells. Moreover, the expression of E-cadherin was also increased, accompanied by decreased expression of Vimentin after transfection of the emmprin siRNA into HEC-50B and KLE cells. Furthermore, the expression of Snail was significantly decreased after transfection of the emmprin siRNA into KLE cells (Figure 5B). 

### NF-κB and AP-1 signal transduction after emmprin siRNA transfection

Emmprin can promote tumor cell invasion via activation of NF-κB and JNK pathways. The activation of AP-1 transcriptional activity may be induced by signaling mediated by JNK and extracellular signal-regulated kinase (ERK) pathways. In the present study, we examined the effects of the emmprin siRNA on the transcriptional activities of NF-κB and AP-1 in HEC-50B and KLE cells. The NF-κB transcriptional activities were decreased after transfection of the emmprin siRNA into HEC-50B and KLE cells. However, the AP-1 transcriptional activities were not decreased after transfection of the emmprin siRNA into HEC-50B and KLE cells (Figure 5C). NF-κB p65 was not affected by transfection of the emmprin siRNA into HEC-50B and KLE cells. However, NF-κB p-p65 was significantly decreased after transfection of the emmprin siRNA into KLE and HEC-251 cells (Figure 5D). NF-κB p50, JNK and ERK were not decreased after transfection of the emmprin siRNA into KLE and HEC-251 cells (data not shown).

### MMP-2, MMP-9 and VEGF expression after emmprin siRNA transfection

VEGF, MMP-2 and MMP-9 are thought to be critically involved in the processes of tumor cell migration, invasion and metastasis. We examined VEGF, MMP-2 and MMP-9 expression after transfection of the emmprin siRNA into HEC-50B and KLE cells. Transfection of the emmprin siRNA caused significant decreases in the expression of MMP-2 and VEGF in HEC-50B and KLE cells. The expression of MMP-9 was significantly decreased by transfection of the emmprin siRNA into KLE cells (Figure 5E).

### Cell growth in monolayers

The effects of emmprin expression on cell proliferation were analyzed after transfection of the emmprin siRNA into the HEC-50B and KLE endometrial cancer cell lines. We found significant inhibitory effects on cell proliferation after transfection of the emmprin siRNA into HEC-50B and KLE cells compared with transfection of the wild-type or empty vector (*p* < 0.05) (Figure 5F).

## Discussion

Proteins on the cell surface play important roles during cancer progression and metastasis via their ability to mediate cell-cell interactions and navigate the communication between cells and the microenvironment. Emmprin is a transmembrane glycoprotein belonging to the immunoglobulin superfamily of molecules expressed on the cell surface of most tumor cells. Several lines of data have clearly shown key roles for emmprin in tumor progression and metastasis. Indeed, high levels of emmprin have been reported in many cancers [11-19]. Accordingly, emmprin expression has been found to be associated with known risk factors for breast cancer and with a poor prognosis of breast cancer patients [19]. In endometrial cancer, Ueda et al. reported that high levels of emmprin expression had a significant association with recurrence [33]. However, the correlation between emmprin expression and survival has not been studied in endometrial cancer. In the present study, we examined whether the emmprin expression levels were associated with uterine normal endometrium, endometrial hyperplasia and endometrial cancer specimens. The levels of emmprin expression were significantly increased in the endometrial cancer specimens relative to the normal endometrium and endometrial hyperplasia specimens. Immunohistochemistry of endometrial cancer specimens from chemosensitivity patients revealed increased emmprin expression with clinicopathological parameters such as FIGO stage, histology, depth of myometrial invasion, cervical involvement, lymph node metastasis, LVS involvement and peritoneal cytology. Moreover, high levels of emmprin expression were significantly associated with the DFS and OS rates in endometrial cancer. Interestingly, these findings indicate that low emmprin expression might be a predictor of favorable prognosis in endometrial cancer patients.

Glycosylated transmembrane proteins are likely to have important roles in cellular homeostasis and their dysregulated activities and expression have been implicated in tumor development and progression. Emmprin plays a role in a variety of physiological processes as is evident by the diverse deficiencies detectable in emmprin knockout mice. Knockout mice deficient in the emmprin gene are sterile and show various neurological abnormalities. emmprin-deficient embryos are also difficult to implant. Emmprin has been found to participate in the cell-surface orientation of monocarboxylic acid transporters (MCTs) to the plasma membrane. Dysfunction of the retina in emmprin-deficient mice is ascribed to the failure of plasma membrane integration of MCTs in the tissue [5]. To date, several studies have suggested possible roles for emmprin in the invasion of carcinoma cells. Emmprin in tumor cells triggers the production or release of matrix metalloproteinases in the surrounding mesenchymal cells and tumor cells, thereby contributing to tumor invasion. Furthermore, the association of emmprin with integrins might be important in signaling through emmprin. Engineered overexpression of emmprin in MDA-MB-231 human breast cancer cells stimulated cell invasion but not cell proliferation [34]. Emmprin regulates cell adhesion, invasion, and cytoskeleton reorganization in prostate cancer cells [35]. Bladder, prostate and gastic cancer, after transfection with emmprin siRNA, showed a significant inhibitory effect of cell proliferation and invasion [36-38]. In the present study, we found the inhibition of cell proliferation, migration and invasion in endometrial cancer cells after transfection of the emmprin siRNA.

The activation of emmprin may be promoted by multiple mechanisms. Emmprin can also interact with key adhesion proteins such as integrins, implying roles in cancer cell migration and invasion. Emmprin is an upstream inducer of several MMPs and is suggested to be the master regulator of MMP production in disease states such as cancer metastasis. In prostate cancer, Emmprin knockdown by the siRNA led to migration and invasion through MMP-2, and MMP-9 expression [38]. The inhibition by emmprin siRNA mediated migration and proliferation, which led to apoptosis by VEGF receptor-2/VEGF system and the matrix degrading protease to block tumor cell growth and invasion in malignant melanoma [39].

EMT, which has been recognized for several decades as being critical for embryogenesis, has also recently been shown to be relevant to cancer progression. Hence, MMP activities have been implicated in EMT by activating growth factors and their receptors and cleaving cell-cell and cell-ECM adhesion molecules. Loss of cell-cell adhesion is a prerequisite for EMT and involves functional loss of E-cadherin. Activation of E-cadherin repressors may be induced by signaling mediated by growth factors, such as TGF-β, EGF, IGF and TNF-α, via their specific receptors. The resultant signaling cascade induces E-cadherin transcription repressors such as Snail, Slug, SIP1 and Twist, eventually resulting in a loss of epithelial morphology [22-29]. Emmprin/CD147 promotes EMT through TGF-β signaling and is transcriptionally regulated by Slug on hepatocellular carcinoma [40]. In this study, we used RT-PCR assays to examine whether transcriptional repressors of growth factors and EMT family were affected in endometrial cancer cells after transfection of the emmprin siRNA. The transfection of the emmprin siRNA caused significant increases in the expression of E-cadherin. The activation of E-cadherin repressors may be induced by emmprin signaling mediated through TGF-β and EGF. Moreover, the expression of E-cadherin was also increased, accompanied by enhanced expression of Vimentin and Snail after transfection of the emmprin siRNA. However, IGF-1, TNF-α, Slug, SIP1 and Twist were unaffected by transfection of the emmprin siRNA (data not shown).

Emmprin can promote tumor cell invasion via activation of NF-κB, JNK, MMPs and VEGF and is thus implicated in other aspects of tumor progression, such as tumor survival and angiogenesis [20,21]. Venkatesan et al. reported that emmprin promoted NF-κB- and AP-1-dependent response gene activation [41]. In this study, we investigated the mechanism of action by the emmprin siRNA in endometrial cancer cell lines. The NF-κB transcriptional activities were decreased after transfection of the emmprin siRNA. Furthermore, NF-κB p-p65 was significantly decreased after transfection of the emmprin siRNA into endometrial cancer cells. However, AP-1 was not affected by transfection of the emmprin siRNA into endometrial cancer cells.

The functions of NF-κB in the transcription of the mesenchymal genes encoding VEGF, Vimentin, MMP-2 and MMP-9 are critical for promoting and maintaining a mesenchymal phenotype in cancers.

Emmprin is involved in cancer development through its ability to stimulate MMP and VEGF production and consequently control extracellular remodeling and anchor-independent growth. MMP-9 and VEGF were induced via the NF-κB pathway in an emmprin-dependent manner in osteotropic breast cancer cells [42]. Emmprin induces osteolysis as a direct emmprin activity as well as the induction of release of downstream effectors, including VEGF and MMP-9 [38]. In this study, we used RT-PCR assays to examine whether VEGF, MMP-2 and MMP-9 were affected in endometrial cancer cells after transfection of the emmprin siRNA. The transfection of the emmprin siRNA caused significant decreases in the expression of VEGF, MMP-2 and MMP-9 in endometrial cancer cells. Overall, the inhibition caused by the emmprin siRNA affected cell proliferation, migration and invasion through TGF-β, EGF, NF-κB, VEGF, MMP-2 and MMP-9 expression, which in turn resulted in increased levels of E-cadherin and reduced levels of Vimentin and Snail in endometrial cancer.

In summary, this study has revealed a critical role for emmprin in endometrial cancer. The present findings suggest that low emmprin expression might be a predictor of favorable prognosis in patients with endometrial cancer, and that emmprin may represent a potential therapeutic target for endometrial cancer.

## Conclusions

The present study has shown that the levels of emmprin expression were significantly increased in the endometrial cancer specimens compared with the normal endometrium and endometrial hyperplasia specimens. High emmprin expression was a significant predictor for a poor prognosis compared with low emmprin expression. Emmprin knockdown by the siRNA led to cell proliferation, migration and invasion through TGF-β, EGF, NF-κB, VEGF, MMP-2, and MMP-9 expression, which in turn resulted in increased levels of E-cadherin and reduced levels of Vimentin and Snail in endometrial cancer. Low emmprin expression might be a predictor of favorable prognosis in endometrial cancer patients, and that emmprin may represent a potential therapeutic target for endometrial cancer.

## Abbreviations

Emmprin: Extracellular matrix metalloproteinase inducer; ECM: Extracellular matrix; NF-κB: Nuclear factor kappa B; JNK: c-Jun N-terminal kinase; VEGF: Vascular endothelial cell growth factor; EMT: Epithelial-mesenchymal transition; TGF: Transforming growth factor; IGF: Insulin-like growth factor; EGF: Epidermal growth factor; TNF: Tumor necrosis factor; SIP1: Smadinteracting protein 1; FIGO: International Federation of Gynecology and Obstetrics; DFS: Disease-free survival; OS: Overall survival; DMEM: Dulbecco’s modified eagle’s medium; FBS: Fetal bovine serum; GAPDH: Glyceraldehyde- 3-phosphate dehydrogenase; ERK: Extracellular signal-regulated kinase.

## Competing interests

The authors declare that they have no competing interests.

## Authors’ contributions

KN contributed to study design, laboratory work, data collection, data management, statistical analysis, data interpretation, and manuscript writing. JK, AH and YH participated in its design and coordination and helped to draft the manuscript. All authors read and approved the final manuscript.

## Acknowledgements

Nothing to declare.

## Pre-publication history

The pre-publication history for this paper can be accessed here:

http://www.biomedcentral.com/1471-2407/12/191/prepub

## References

[B1] Jemal A Siegel R Xu J Ward E Cancer statistics, 2010 CA Cancer J Clin 2010 60 277 300 10.3322/caac.20073 20610543

[B2] Annual Report of Oncology Committee of Japan Society of Obstetrics and Gynecology Acta Obstet et gynaecol Japon 2012 64 1043 1054 Japanese.

[B3] Biswas C Zhang Y DeCastro R Guo H Nakamura T Kataoka H Nabeshima K The human tumor cell-derived collagenase stimulatory factor (renamed EMMPRIN) is a member of the immunoglobulin superfamily Cancer Res 1995 55 434 439 7812975

[B4] Guo H Majmudar G Jensen TC Biswas C Toole BP Gordon MK Characterization of the gene for human EMMPRIN, a tumor cell surface inducer of matrix metalloproteinases Gene 1998 220 99 108 10.1016/S0378-1119(98)00400-4 9767135

[B5] Muramatsu T Miyauchi T Basigin (CD147): a multifunctional transmembrane protein involved in reproduction, neural function, inflammation and tumor invasion Histol Histopathol 2003 18 981 987 12792908 10.14670/HH-18.981

[B6] Chen X Kanekura T Kanzaki T Expression of Basigin in human fetal, infantile and adult skin and in basal cell carcinoma J Cutan Pathol 2001 28 184 190 10.1034/j.1600-0560.2001.028004184.x 11426825

[B7] Gabison EE Hoang-Xuan T Mauviel A Menashi S EMMPRIN/CD147, an MMP modulator in cancer, development and tissue repair Biochimie 2005 87 361 368 10.1016/j.biochi.2004.09.023 15781323

[B8] Zhu P Ding J Zhou J Dong WJ Fan CM Chen ZN Expression of CD147 on monocytes/macrophages in rheumatoid arthritis: its potential role in monocyte accumulation and matrix metalloproteinase production Arthritis Res Ther 2005 7 1023 1033 10.1186/ar1778 PMC1257431 16207318

[B9] Nabeshima K Iwasaki H Koga K Hojo H Suzumiya J Kikuchi M Emmprin (basigin/CD147): matrix metalloproteinase modulator and multifunctional cell recognition molecule that plays a critical role in cancer progression Pathol Int 2006 56 359 367 10.1111/j.1440-1827.2006.01972.x 16792544

[B10] Yan L Zucker S Toole BP Roles of the multifunctional glycoprotein, emmprin (basigin; CD147), in tumour progression Thromb Haemost 2005 93 199 204 15711733 10.1160/TH04-08-0536

[B11] Jin JS Yao CW Loh SH Cheng MF Hsieh DS Bai CY Increasing expression of extracellular matrix metalloprotease inducer in ovary tumors: tissue microarray analysis of immunostaining score with clinicopathological parameters Int J Gynecol Pathol 2006 25 140 146 10.1097/01.pgp.0000189244.57145.84 16633062

[B12] Sameshima T Nabeshima K Toole BP Yokogami K Okada Y Goya T Koono M Wakisaka S Expression of emmprin (CD147), a cell surface inducer of matrix metalloproteinases, in normal human brain and gliomas Int J Cancer 2000 88 21 27 10.1002/1097-0215(20001001)88:1<21::AID-IJC4>3.0.CO;2-S 10962435

[B13] Zheng HC Takahashi H Murai Y Cui ZG Nomoto K Miwa S Tsuneyama K Takano Y Upregulated EMMPRIN/CD147 might contribute to growth and angiogenesis of gastric carcinoma: a good marker for local invasion and prognosis Br J Cancer 2006 95 1371 1378 10.1038/sj.bjc.6603425 17088917 PMC2360592

[B14] Bordador LC Li X Toole B Chen B Regezi J Zardi L Hu Y Ramos DM Expression of emmprin by oral squamous cell carcinoma Int J Cancer 2000 85 347 352 10.1002/(SICI)1097-0215(20000201)85:3<347::AID-IJC9>3.0.CO;2-# 10652425

[B15] Kanekura T Chen X Kanzaki T Basigin (CD147) is expressed on melanoma cells and induces tumor cell invasion by stimulating production of matrix metalloproteinases by fibroblasts Int J Cancer 2002 99 520 528 10.1002/ijc.10390 11992541

[B16] Li Y Xu J Chen L Zhong WD Zhang Z Mi L Zhang Y Liao CG Bian HJ Jiang JL Yang XM Li XY Fan CM Zhu P Fu L Chen ZN HAb18G (CD147), a cancer-associated biomarker and its role in cancer detection Histopathology 2009 54 677 687 10.1111/j.1365-2559.2009.03280.x 19438743

[B17] Zhang Q Zhou J Ku XM Chen XG Zhang L Xu J Chen GS Li Q Qian F Tian R Wen N Chen ZN Expression of CD147 as a significantly unfavorable prognostic factor in hepatocellular carcinoma Eur J Cancer Prev 2007 16 196 202 10.1097/01.cej.0000236245.40619.c3 17415090

[B18] Tan H Ye K Wang Z Tang H CD147 expression as a significant prognostic factor in differentiated thyroid carcinoma Transl Res 2008 152 143 149 10.1016/j.trsl.2008.07.005 18774544

[B19] Reimers N Zafrakas K Assmann V Egen C Riethdorf L Riethdorf S Berger J Ebel S Jänicke F Sauter G Pantel K Expression of extracellular matrix metalloproteases inducer on micrometastatic and primary mammary carcinoma cells Clin Cancer Res 2004 10 3422 3428 10.1158/1078-0432.CCR-03-0610 15161697

[B20] Hagemann T Wilson J Kulbe H Li NF Leinster DA Charles K Klemm F Pukrop T Binder C Balkwill FR Macrophages induce invasiveness of epithelial cancer cells via NF-kappa B and JNK J Immunol 2005 175 1197 1205 16002723 10.4049/jimmunol.175.2.1197

[B21] Tang Y Nakada MT Kesavan P McCabe F Millar H Rafferty P Bugelski P Yan L Extracellular matrix metalloproteinase inducer stimulates tumor angiogenesis by elevating vascular endothelial cell growth factor and matrix metalloproteinases Cancer Res 2005 65 3193 3199 15833850 10.1158/0008-5472.CAN-04-3605

[B22] Yang J Weinberg RA Epithelial-mesenchymal transition: at the crossroads of development and tumor metastasis Dev Cell 2008 14 818 829 10.1016/j.devcel.2008.05.009 18539112

[B23] Christiansen JJ Rajasekaran AK Reassessing epithelial to mesenchymal transition as a prerequisite for carcinoma invasion and metastasis Cancer Res 2006 66 8319 8326 10.1158/0008-5472.CAN-06-0410 16951136

[B24] Berx G Raspé E Christofori G Thiery JP Sleeman JP Pre-EMTing metastasis? Recapitulation of morphogenetic processes in cancer Clin Exp Metastasis 2007 24 587 597 10.1007/s10585-007-9114-6 17978854

[B25] Moustakas A Heldin CH Signaling networks guiding epithelial -mesenchymal transitions during embryogenesis and cancer progression Cancer Sci 2007 98 1512 1520 10.1111/j.1349-7006.2007.00550.x 17645776 PMC11158989

[B26] Batlle E Sancho E Francí C Domínguez D Monfar M Baulida J García De Herreros A The transcription factor snail is a repressor of E-cadherin gene expression in epithelial tumour cells Nat Cell Biol 2000 2 84 89 10.1038/35000034 10655587

[B27] Cano A Pérez-Moreno MA Rodrigo I Locascio A Blanco MJ del Barrio MG Portillo F Nieto MA The transcription factor snail controls epithelial -mesenchymal transitions by repressing E-cadherin expression Nat Cell Biol 2000 2 76 83 10.1038/35000025 10655586

[B28] Hajra KM Chen DY Fearon ER The SLUG zinc-finger protein represses E-cadherin in breast cancer Cancer Res 2002 62 1613 1618 11912130

[B29] Yang J Mani SA Donaher JL Ramaswamy S Itzykson RA Come C Savagner P Gitelman I Richardson A Weinberg RA Twist, a master regulator of morphogenesis, plays an essential role in tumor metastasis Cell 2004 117 927 939 10.1016/j.cell.2004.06.006 15210113

[B30] Li M Zhai Q Bharadwaj U Wang H Li F Fisher WE Chen C Yao Q Cyclophilin A is overexpressed in human pancreatic cancer cells and stimulates cell proliferation through CD147 Cancer 2006 106 2284 2294 10.1002/cncr.21862 16604531

[B31] Soulitzis N Karyotis I Delakas D Spandidos DA Expression analysis of peptide growth factors VEGF, FGF2, TGFB1, EGF and IGF1 in prostate cancer and benign prostatic hyperplasia Int J Oncol 2006 29 305 314 16820871

[B32] Cheng H Fukushima T Takahashi N Tanaka H Kataoka H Hepatocyte growth factor activator inhibitor type 1 regulates epithelial to mesenchymal transition through membrane-bound serine proteinases Cancer Res 2009 69 1828 1835 10.1158/0008-5472.CAN-08-3728 19223533

[B33] Ueda K Yamada K Urashima M Ishibashi Y Shirai M Nikaido T Takahashi H Okamoto A Saito M Yasuda M Ohkawa K Tanaka T Association of extracellular matrix metalloproteinase inducer in endometrial carcinoma with patient outcomes and clinicopathogenesis using monoclonal antibody 12C3 Oncol Rep 2007 17 731 735 17342307

[B34] Rucci N Millimaggi D Mari M Del Fattore A Bologna M Teti A Angelucci A Dolo V Receptor activator of NF-kappaB ligand enhances breast cancer-induced osteolytic lesions through upregulation of extracellular matrix metalloproteinase inducer/CD147 Cancer Res 2010 70 6150 6160 10.1158/0008-5472.CAN-09-2758 20631064

[B35] Zhu H Zhao J Zhu B Collazo J Gal J Shi P Liu L Ström AL Lu X McCann RO Toborek M Kyprianou N EMMPRIN regulates cytoskeleton reorganization and cell adhesion in prostate cancer Prostate 2012 72 72 81 10.1002/pros.21408 21563192 PMC3158271

[B36] Xue YJ Lu Q Sun ZX CD147 overexpression is a prognostic factor and a potential therapeutic target in bladder cancer Med Oncol 2011 28 1363 1372 10.1007/s12032-010-9582-4 20509007

[B37] Wang B Xu YF He BS Pan YQ Zhang LR Zhu C Qu LL Wang SK RNAi-mediated silencing of CD147 inhibits tumor cell proliferation, invasion and increases chemosensitivity to cisplatin in SGC7901 cells in vitro J Exp Clin Cancer Res 2010 29 61 10.1186/1756-9966-29-61 20525232 PMC2893454

[B38] Wang L Wu G Yu L Yuan J Fang F Zhai Z Wang F Wang H Inhibition of CD147 expression reduces tumor cell invasion in human prostate cancer cell line via RNA interference Cancer Biol Ther 2006 5 608 614 10.4161/cbt.5.6.2661 16627983

[B39] Bougatef F Menashi S Khayati F Naïmi B Porcher R Podgorniak MP Millot G Janin A Calvo F Lebbé C Mourah S EMMPRIN promotes melanoma cells malignant properties through a HIF-2alpha mediated up-regulation of VEGF-receptor-2 PLoS One 2010 5 e12265 10.1371/journal.pone.0012265 20824203 PMC2930842

[B40] Wu J Ru NY Zhang Y Li Y Wei D Ren Z Huang XF Chen ZN Bian H HAb18G/CD147 promotes epithelial-mesenchymal transition through TGF-β signaling and is transcriptionally regulated by Slug Oncogene 2011 30 4410 4427 10.1038/onc.2011.149 21532623

[B41] Venkatesan B Valente AJ Prabhu SD Shanmugam P Delafontaine P Chandrasekar B EMMPRIN activates multiple transcription factors in cardiomyocytes, and induces interleukin-18 expression via Rac1-dependent PI3K/Akt/IKK/NF-kappaB andMKK7/JNK/AP-1 signaling J Mol Cell Cardiol 2010 49 655 663 10.1016/j.yjmcc.2010.05.007 20538003 PMC3042694

[B42] Min C Eddy SF Sherr DH Sonenshein GE NF-kappaB and epithelial to mesenchymal transition of cancer J Cell Biochem 2008 104 733 744 10.1002/jcb.21695 18253935

